# An engineered food-grade *Lactococcus lactis* strain for production and delivery of heat-labile enterotoxin B subunit to mucosal sites

**DOI:** 10.1186/s12896-017-0345-6

**Published:** 2017-03-06

**Authors:** Nan Sun, Rongguang Zhang, Guangcai Duan, Xiaoyan Peng, Chen Wang, Qingtang Fan, Shuaiyin Chen, Yuanlin Xi

**Affiliations:** 1 Department of Epidemiology and Statistics, College of Public Health, Zhengzhou University, Zhengzhou, 450001 China; 2 Henan Innovation Center of Molecular Diagnosis and Laboratory Medicine, Xinxiang Medical University, Xinxiang, 453003 China

**Keywords:** *Helicobactor pylori*, *Lactococcus lactis*, Lpp20, Food-grade, Oral vaccine, LTB

## Abstract

**Background:**

Recent researches have been focusing on mucosal immune adjuvants, which play the key roles in mucosal immunization and have become the limitation for non-injected vaccine development. *Escherichia coli* heat-labile enterotoxin B subunit (LTB) was regarded as a promising mucosal adjuvant for its nontoxicity and potent activity. LTB preparation issues have always been recurring, in part owing to that the recombinant LTB expressed by *E. coli* does not act as its native form.

**Results:**

We constructed an engineered *Lactococcus lactis* strain using a food-grade expression system. The LTB secreted by the engineered strain was detected in the culture supernatant, constituting 10.3% of the supernatant proteins, and recognized by mouse anti-LTB antibodies. The engineered strain, co-administered orally to SPF BALB/c mice with a *H. pylori* vaccine candidate expressing Lpp20 antigen, could significantly enhance the Lpp20-induced mucosal SIgA antibody responses against *H. pylori*.

**Conclusions:**

This is the first report that LTB was efficiently produced and delivered via using a food-grade lactococcal expression system, which offers a novel production and utilization mode of this crucial mucosal adjuvant. The engineered *L. lactis* strain secreting LTB has considerable potential for oral vaccine formulation owing to its outstanding safety, adjuvant activity and high-level production.

**Electronic supplementary material:**

The online version of this article (doi:10.1186/s12896-017-0345-6) contains supplementary material, which is available to authorized users.

## Highlights

1. This is the first report that LTB was efficiently produced and delivered via using a food-grade lactococcal expression system.
2. This study offers a novel production and utilization mode of this crucial mucosal adjuvant.
3. The engineered *L. lactis* strain secreting LTB has considerable potential for oral vaccine formulation owing to its outstanding safety, adjuvant activity and high-level production.

## Background

Gastroenteric infections cause an estimated two million deaths worldwide per year, and remain severe public health issues [1, 2]. As antibiotic resistance has been continually increasing, researches currently focus on developing vaccines against the causative agents, such as *Helicobacter pylori*, Shigella and enterotoxigenic *E. coli* (ETEC), for which no commercial vaccines are available [1].

Presently, most commercial vaccines are administered by parenteral routes [3]. However, recent studies demonstrated mucosal vaccination as the most effective strategy against the pathogens that colonize or invade mucosa to initiate lesions [3–5]. Although parenteral immunizations can protect against causative agents parasitizing host tissues via stimulation of serum antibody and cellular immune responses, they can hardly elicit mucosal immunity against noninvasive pathogens [3, 6]**.** Mucosal vaccination can stimulate secretory antibody responses preventing infection by the pathogens from the mucosal surface [5]. Additionally, mucosal immunizations have the advantages of simple manipulation, less invasion, lowered risks of disease transmissions and ease of manufacture over parenteral inoculations.

However, mucosal vaccinations with antigens alone are commonly insufficient to induce marked immune responses, unless the antigens can reach the mucosal inductive sites as cholera toxins [3, 7]. As proved, mucosal adjuvants or microbial delivery vectors are required for effective mucosal immune responses [8]. Therefore, recent researches have emphasized screening and preparation of adjuvants and the biotic delivery vehicles which possess adjuvant activity [3].

*E. coli* heat-labile enterotoxin B subunit (LTB) is a promising mucosal adjuvant, owing to its nontoxicity and potent mucosal adjuvant activity [9]. Nevertheless, LTB preparation issues have always been existing because it is impractical to purify LTB from ETEC for production of vaccines, and the activity of recombinant LTB (rLTB) was greatly affected by the expression hosts employed. Previous studies have indicated that preparation of LTB by using a *E. coli* expression system are not only inefficient but also costly [6]. The reasons involve the recurring formation of insoluble inclusion bodies, lower yields of bioactive rLTB, the cost of protein purification and the risk of pollution with unbeneficial bacterial components like lipopolysaccharide.

To address these issues, such bacteria as attenuated pathogens and probiotics have been exploited as expression hosts and live vectors for LTB production and delivery. A study compared expression efficacy of rLTB in *Pichia pastoris* with that in *E. coli*, demonstrating that a higher expression level and adjuvant activity of rLTB could be obtained by using *P. pastoris* as the expression host [6]. Another study proved that LTB expressed in fusion with antigens in engineered *Lactobacillus* can significantly enhance the local and systemic immune responses to the antigens [10, 11]. Recently, increasing evidences supported that food-grade *L. lactis* expression systems, through expression and delivery of antigens/adjuvants, are promising oral vaccine vectors, particularly owing to their outstanding safety, avoidance of protein purification, reduced antigen degradation and efficient delivery of immunogens to the mucosal inductive sites [12, 13]. However, successful expression of LTB in food-grade *L. lactis* has not been reported to date. Therefore, construction of a food-grade *L. lactis* strain producing LTB should be a considerable step toward the goal of effective and safe mucosal vaccines.

In this work, a food-grade *L. lactis* strain was genetically engineered for production and delivery of LTB, and its immune adjuvant activity was evaluated by oral vaccination of mice with the engineered strain and a Lpp20-based *H. pylori* vaccine candidate. The observations of this study demonstrate a novel efficient production and utilization mode of LTB, which forms a crucial basis for mucosal vaccine formulation.

## Methods

### Bacterial strains, plasmids and growth conditions

The bacteria and plasmids are shown in Additional file 1. The bacterial cultivation conditions were as described previously [12, 14]. SPF BALB/c mice, aged 6 weeks, were purchased from Henan Experimental Animal Center (Zhengzhou, China).

### Polymerase chain reaction of *ltB* gene

The *ltB* gene was amplified by PCR from the plasmid pMAL-c2x-*mlt63* using Pyrobest DNA polymerase (TaKaRa, China). A pair of oligonucleotide primers was designed based on the published sequence (GenBank EF057802). The sequences of the primers were 5’-CAGTCG**GCATGC**GCTCCCCAGTCTATTAC-3’ (Sense) and 5’-CGC**TCTAGA**CTAGTTTTCCATACTGATTG-3’ (Antisense) with the endonuclease sites *Sph*I and *Xba*I shown in bold letters, respectively. The PCR profile included 30 cycles of 94 °C for 1 min, 55 °C for 30 s and 72 °C for 2 min.

### Construction of recombinant *L. lactis* strains

The *ltB* gene was ligated with TA cloning vector pMD19-T and used to transform *E. coli* DH5α by heat shock. The *ltB* gene fragment was collected by digestion of pMD19-T-*ltB* using *Sph*I and *Xba*I (TaKaRa, China), ligated with plasmid pNZ8149-SP (GenBank KY385376)*,* and used for transformation of *L. lactis* NZ3900 by electrophoration [12]. The recombinants were obtained by Elliker medium selection, and identified by restriction digestion and gene sequencing as reported before [12]. The recombinant NZ3900 strain carrying *ltB* gene was referred to as *L. lactis* NZ3900/pNZ8149-SP-*ltB*.

### LTB expression and western blotting assays

The expression of LTB in NZ3900/pNZ8149-SP-*ltB* was induced using 25 ng/ml nisin (Sigma, USA) as inducer under the conditions as previously described [12].

Samples of the culture supernatant were prepared from 50 ml of culture. The supernatant was obtained by centrifugation at 10,000 rpm for 20 min at 4 °C, and filtered through 0.22 μm filter. The proteins in the filtrate were precipitated by adding trichloroacetic acid (10%, v/v), incubating at 4 °C for 16 h and centrifugation at 10,000 rpm for 30 min at 4 °C. The pellet was resuspended in 8 ml acetone, centrifugated at 10,000 rpm for 20 min at 4 °C, then kept in fume hood at room temperature until dry. The protein sample was added 360 μl PBS, kept at 4 °C for 3 h, and centrifugated at 10,000 rpm 4 °C for 10 min. The supernatant was collected and used as samples of the culture supernatant. Samples of bacterial cell lysates were processed as described before [12].

SDS-PAGE and western blotting assays were performed using mouse anti-LTB antibody (Abcam, USA) as the primary antibody as previously reported [12].

### Oral vaccination of mice

The mice were assigned at random into three groups of 10 each. For Lpp20 group and Lpp20 + LTB group, the mice were treated by gavage with cell suspensions at a dose of 300 μl of NZ3900/pNZ8149-SP-*lpp20* (1 × 10^11^ CFU/ml) and a mixture of NZ3900/pNZ8149-SP-*lpp20* (1 × 10^11^ CFU/ml) and NZ3900/pNZ8149-SP-*ltB* (1 × 10^11^ CFU/ml), respectively, on day 0, 7, 14, 21, 28 and 35. For PBS group, mice were given equal volumes of PBS instead of the cell suspensions.

### Blood and intestinal feces sampling

Seven days after the last vaccination, blood and intestinal feces were collected for half number of the mice from all the groups. The blood samples were fetched from orbital sinus and kept at 4 °C for 16–20 h, and then the sera were separated and stored in aliquots at −20 °C. For sampling intestinal feces, the mice were sacrificed by spinal dislocation, 100 mg of feces was fetched from the intestine for each mouse, and then 1 ml of PBS containing proteinase inhabitor (Phenylmethanesulfonyl fluoride, 0.1 mM) was injected by the duodenum to wash the intestinal wall. The eluate was recovered, mixed with the feces and kept at 4 °C for overnight (14–16 h). The supernatant of the mixture was separated via centrifugation at 12,000 rpm for 10 min, and stored at −20 °C as ELISA samples.

### ELISA detection of IgG and SIgA antibodies

The *H. pylori*-specific serum IgG and fecal SIgA antibodies were quantified by ELISA as described before [12]. Briefly, 96-well microplates (Beijing Solarbio, China) were coated with soluble *H. pylori* somatic proteins. The ELISA signals were developed using biotinylated goat anti-mouse IgG (Abcam, USA), goat anti-mouse SIgA (Abcam, USA) and p-nitrophenyl phosphate (PNPP) substrate (Beijing Solarbio, China). The absorbances of the wells at 450 nm (OD_450_) were measured using a Microplate Reader (Tecan Sunrise, CH), and designed as indicators of the specific sIgA and IgG levels.

### Statistical analysis

The measurement data were presented as means ± standard deviation $\left(\overline{x},\pm, s\right).$ The significance of the difference among the groups was tested using Kruskal-Wallis tests, while the pairwise comparisons of mean values were carried out using the Mann-Whitney U test with the aid of software SAS9.13. The difference was considered as significant at *P* <0.05.

## Results

### Genetic engineering of *L. lactis*

**Fig. 1:**
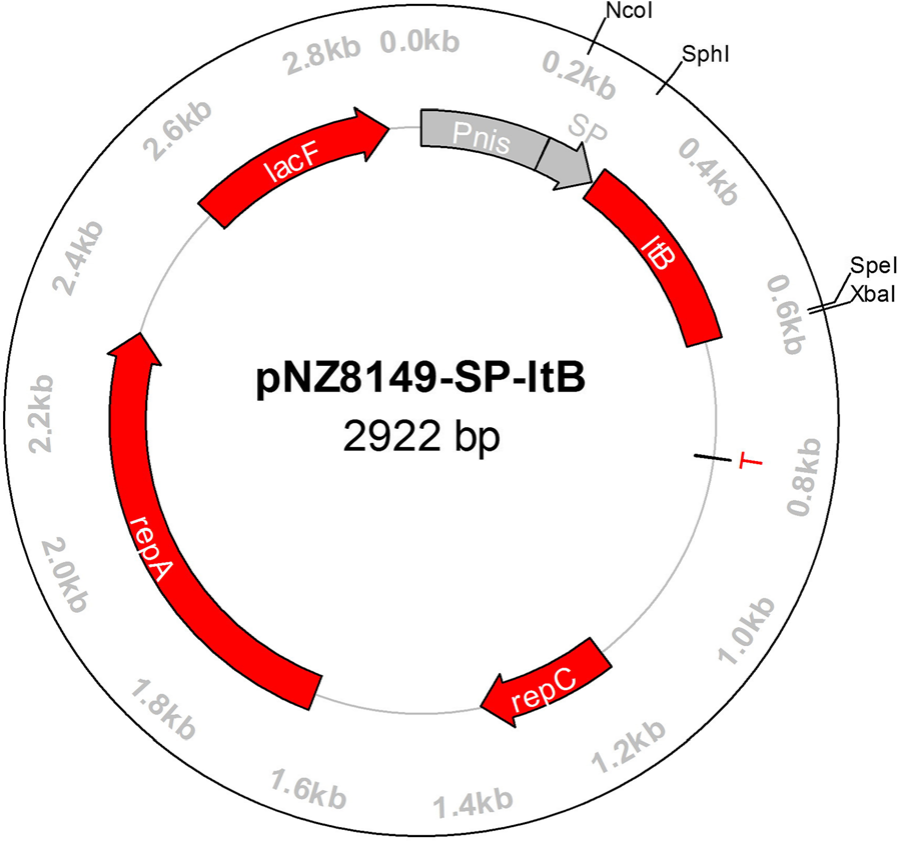
The schematic maps of pNZ8149-SP-*ltB* designed using software Omiga 2.0. Pnis, nisA promoter; SP, signal sequence of *usp*45 gene; ltB, *ltB* gene coding region; T, terminator; repA and repC, replication gene A and C; lacF, *lacF* gene as selection signature

The PCR product of *ltB* gene was 334 bp in length, and 100% identical in nucleotide sequence to the published *E. coli ltB* gene (GenBank JX504011.1). The plasmids were isolated from the *L. lactis* transformants, and identified by restriction enzyme digestion. Gene sequencing confirmed that the *ltB* gene was exactly cloned downstream of the nisin controlled promoter (*Pnis*) within the expression vector pNZ8149-SP, generating pNZ8149-SP-*ltB*. The schematic diagram of pNZ8149-SP*-ltB* was shown in Fig. 1.

### Expression and immunoreactivity of Lpp20

**Fig. 2:**
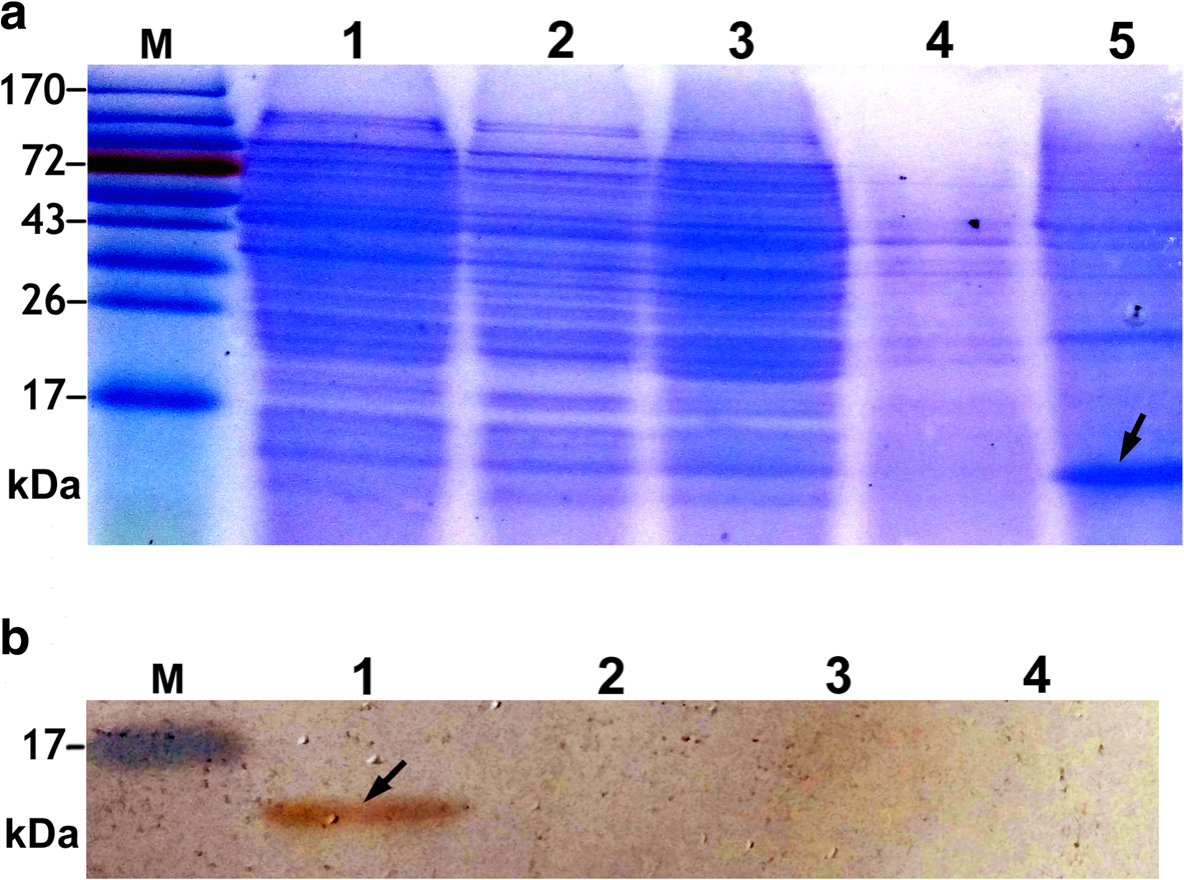
Analysis of cell lysate and culture supernatant proteins of the nisin-induced *L. lactis* strains. The arrows indicate the expressed UreB. **a** SDS-PAGE. Lane M, protein markers; lane 1, 2, 3, cell lysates of NZ3900, NZ3900/pNZ8149-SP and NZ3900/pNZ8149-SP-*ltB*, respectively; lane 4, 5, culture supernatant of NZ3900/pNZ8149-SP and NZ3900/pNZ8149-SP-*ltB*, respectively. These figures showed that the recombinant LTB was expressed as a secreted protein. **b** Western blotting assays. *L. lactis* NZ3900/pNZ8149-SP was used as the negative control, while mouse anti-LTB antibodies as the primary antibodies for detection of LTB. Lane M, protein markers; lane 1, 2, culture supernatant of NZ3900/pNZ8149-SP-*ltB* and NZ3900/pNZ8149-SP, respectively; lane 3, 4, cell lysates of NZ3900/pNZ8149-SP-*ltB* and NZ3900/pNZ8149-SP, respectively. These figures showed that the expressed LTB was detectable only in the culture supernatant of NZ3900/pNZ8149-SP-*ltB,* and possessed immunoreactivity with the commercial anti-LTB antibody

To determine inducible expression of LTB in *L. lactis*, the cell lysates and culture supernatant were analyzed by SDS-PAGE and western blots. As results, SDS-PAGE analysis showed that a dense protein band was present at approximately 13 kDa position in the culture supernatant of NZ3900/pNZ8149-SP-*ltB*, while absent at the corresponding position in the controls (Fig. 2a). The percentage of LTB reached 10.3% in the culture supernatant proteins of the engineered stain.

Western blotting analysis showed that the 13 kDa protein in the culture supernatant of NZ3900/pNZ8149-SP-*ltB* could be recognized by the mouse anti-LTB antibodies, while there was no positive band at the corresponding position in the lanes of NZ3900/pNZ8149-SP samples as negative controls (Fig. 2b).

### Immunization of mice and antibody assays

**Fig. 3:**
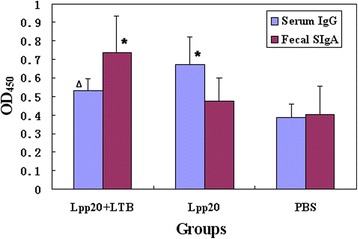
OD_450_ values of ELISA tests for *H. pylori*-specific serum IgG and intestinal SIgA levels. The Lpp20 group and Lpp20 + LTB group were vaccinated with *L. lactis* NZ3900/pNZ8149-SP-*lpp20* and the mixture of NZ3900/pNZ8149-SP-*lpp20 and* NZ3900/pNZ8149-SP-*ltB*, respectively, while the PBS group, as the control, was treated with PBS. One week after oral immunization, blood sampling was performed for half number of the mice of all the groups. The serum (1:10 diluted) and fecal samples were tested for *H. pylori*-specific antibodies by ELISA. Bars, mean OD_450_ value; error bars, standard deviation; ^∆^ the value was significantly higher than that for the PBS group (*P* < 0.05); * the value was significantly different from those for the other two groups (*P* < 0.05)

The *L. lactis* NZ3900/pNZ8149-SP-*lpp20 and* NZ3900/pNZ8149-SP-*ltB* were induced with nisin as mentioned above. The mice of the immunized groups were orally inoculated with the induced NZ3900/pNZ8149-SP-*lpp20* alone and its mixture with NZ3900/pNZ8149-SP-*ltB*, respectively, while the control group was treated with PBS. On day 7 after the final vaccination, the serum and intestinal feces were sampled, and tested for *H. pylori*-specific antibodies by ELISA assays. The results are shown in Additional file 2 and Fig. 3. The group treated with NZ3900/pNZ8149-SP-*lpp20* alone had enhanced anti-*H. pylori* serum antibody responses, but no significantly elevated intestinal SIgA level, compared with the PBS group. The vaccination with the mixture of the two *L. lactis* strains evoked significantly elevated SIgA antibody level, and insignificantly lowered the serum IgG response, as compared with the group administered with NZ3900/pNZ8149-SP-*lpp20* alone. The results demonstrated the adjuvant activity of the engineered *L. lactis* expressing LTB to promote mucosal immune responses.

## Discussion

As proved, the species of bacteria as expression hosts might greatly affect the bioactivity of recombinant expression products [6]. Through screening of various microbial expression systems, it is possible to establish an efficient method for production and utilization of LTB. Nevertheless, although studies have demonstrated the potential of LTB as mucosal adjuvant and accumulated evidences proved *L. lactis* to be a safe vaccine vehicle, expression and delivery of LTB using a food-grade *L. lactis* strain have not been reported yet. Therefore, successfully engineering a food-grade *L. lactis* strain to secret LTB can be a crucial basis for mucosal vaccine development.

*L. lactis* NZ3900/pNZ8149-SP is a food-grade nisin-controlled expression system, which was constructed in our previous study by introducing a gene fragment encoding the signal peptide of *L. lactis* Usp45 protein into pNZ8149 for capacity of secretory expression of heterologous genes [12]. In this work, the *ltB* gene was inserted downstream of the nisin controlled promoter within pNZ8149-SP, and expression of LTB was achieved by inducement with food-grade subtoxic amount of nisin. All the components of *L. lactis* NZ3900/pNZ8149-SP*-ltB*, except for the target gene *ltB*, possess food-grade safety, suggesting the extremely high safety of the engineered strain as the LTB producer.

As reported, the *L. lactis* expression efficiency of heterologous protein can be rather low, and thus the expression products in certain studies were detectable by western blots, but not by SDS-PAGE [15, 16]. In this work, the expressed LTB was visual as the predominant band in the SDS-PAGE pattern, constituting 10.3% of the culture supernatant extracts of the engineered *L. lactis*. The expression efficacy was much higher than those reported [12, 15–17]. The mechanism underlying this phenomenon might include the diversity of the heterologous genes in genetic codon constitution. The LTB expressed in *L. lactis* had a molecular weight of approximately 13 kDa, corresponding to that deduced from the nucleotide sequence (GenBank JX504011.1). Western blotting assays showed that LTB produced by the engineered *L. lactis* strain retained potent antigenicity. These findings indicate that the engineered strain culture can be a novel source of LTB, from which LTB can be obtained and utilized without protein purification and the risk of pollution with toxic components from the commonly used expression hosts, such as *E. coli* and *Salmonella spp.*.

A recurring issue on heterogeneous expression is that the recombinant products might lose bioactivity of the native proteins. Although the molecular basis of LTB adjuvant properties remains unclear, it might be included that LTB interacts with such receptors as GM1-ganglioside accelerating uptake of the toxic subunit A into the epithelial cells [6, 18]. In the present study, it was observed that vaccination of mice with *L. lactis* expressing Lpp20 was capable of arousing remarkable serum IgG antibody responses, but failed to induce significantly elevated intestinal specific SIgA antibody levels, as compared with the group given PBS, indicating that without the aid of suitable mucosal adjuvants, the employed *L. lactis* strain producing Lpp20 can hardly evoke notable mucosal immune responses. Similarly, a previous study showed that a *L. lactis* strain producing *H. pylori* UreB antigen was unable to elicit marked immune responses in mice [17]. The adjuvant activity of the engineered strain secreting LTB has been proved by the observations that the group received the mixture of the two engineered *L. lactis* strains expressing Lpp20 and LTB, respectively, had significantly elevated sIgA antibodies, in comparison with the group given the Lpp20-producing strain alone. Since mucosal SIgA antibodies have the key roles in immune protection against the pathogens invading human tissues by the mucosal surface, the findings of our study suggest that the engineered strain secreting LTB might markedly enhance the immune protective efficacy of the co-administered antigens [19].

Interestingly, accompanying with the enhanced mucosal SIgA, the serum IgG antibodies seemed to be suppressed in the mice immunized with the mixture of the two *L. lactis* strains. This result suggests that the LTB-secreting *L. lactis* might modulate the systemic and mucosal immune responses with opposite effects. The mechanism and its implication for vaccine designs need further investigation.

In recent decades, although in most countries the use of genetically modified organisms (GMOs) was prohibited or heavily regulated, it has been proposed to change the present regulations [20, 21]. In fact, the amount of commercially available GMOs has been continuously rising in recent years [22]. It is hopeful for the food-grade *L. lactis* engineered here to be used in pharmacy and food industry in future.

## Conclusion

Taken together, this is the first report that *E. coli* LTB was efficiently produced and delivered via using a food-grade lactococcal expression system, which offers a novel production and utilization mode of this crucial mucosal adjuvant. The engineered *L. lactis* strain secreting LTB has considerable potential for oral vaccine formulation, owing to its outstanding safety, adjuvant activity and high-level production.

## Additional files

Additional file 1: Table S1. Bacteria and plasmids used in this work. (DOC 39 kb)
Additional file 2: Table S2. OD_450_ values of ELISA tests for *H. pylori*-specific IgG and SIgA levels. (DOC 60 kb)

## Abbreviations

ELISA: Enzyme-linked immunosorbent assay
ETEC: Enterotoxigenic *E. coli*
LTB: Heat-labile enterotoxin B subunit
PBS: Phosphate buffer saline
PNPP: P-nitrophenyl phosphate
SPF: Specific pathogens free
UreB: Urease B subunit
