# Supplementary material for: An engineered food-grade Lactococcus lactis strain for production and delivery of heat-labile enterotoxin B subunit to mucosal sites
Source: BMC Biotechnol. 2017 Mar 6;17:25. doi: 10.1186/s12896-017-0345-6 (PMC5339977; doi:10.1186/s12896-017-0345-6)
Supplement: Additional file 1: Table S1. — Bacteria and plasmids used in this work. (DOC 39 kb) [file 12896_2017_345_MOESM1_ESM.doc]

**Table S1.** Bacteria and plasmids used in this work

| Plasmids/Bacteria | Characteristics | Reference/Source |
| --- | --- | --- |
| pMD19-T | A TA cloning vector containing the ampicillinum resistance gene for selection. | TaKaRa, China |
| pMD19-T-*ltB* | pMD19-T carrying *ltB* gene | This study |
| pNZ8149-SP | *L. lactis* NZ3900 secretory expression vector with a signal sequence of *L. lactis* Usp45 protein inserted between *Nco*I and *Sal*I site in the plasmid pNZ8149 (NIZO Food Research, Netherlands), food grade with the *lacF* gene as selection marker | China Patent (No. ZL 2012 1 0218281.9) |
| pNZ8149-SP-*ltB* | pNZ8149-SP carrying *ltB* gene | This study |
| pMAL-c2x-*mlt63* | pMAL-c2x (NEB Co., UK) carrying the mutant derivative *mlt63* of *E. coli* heat-labile toxin encoding gene | (Huang *et al.*, 2013) |
| *L. lactis* NZ3900 | Derivatives of *L. lactis* subsp. cremoris MG1363, *lacF^-^*, pepN::nisRnisK, plasmid-free, food grade | NIZO Food Research, Netherlands |
| *L. lactis* NZ3900 /pNZ8149-SP-*ltB* | *L. lactis* NZ3900 harboring pNZ8149-SP-*ltB* | This study |
| *L. lactis* NZ3900 /pNZ8149-SP-*lpp20* | An engineered *L. lactis* strain constructed using the same method as that for NZ3900/pNZ8149 -SP-*ltB* except using the *lpp20* gene (GenBank KX686594) instead of the *ltB* gene. | Unpublished study |
| *H. pylori* MEL-Hp27 | *cagA*+, *vagA*+, a clinical strain isolated from a Chinese patient with chronic gastritis. | CGMCC* No.1338 |
| *H. pylori* 11637 | *vacA*+, *cagA*+, *H. pylori* line NCTC11637 | Our laboratory |
| *E. coli* DH5α | F-, φ80dlacZΔM15, recA1, Δ(lacZYA-argF), etc. | TaKaRa, China |

* China General Microbiological Culture Collection Center (CGMCC).
