# Supplementary material for: An engineered food-grade Lactococcus lactis strain for production and delivery of heat-labile enterotoxin B subunit to mucosal sites
Source: BMC Biotechnol. 2017 Mar 6;17:25. doi: 10.1186/s12896-017-0345-6 (PMC5339977; doi:10.1186/s12896-017-0345-6)
Supplement: Additional file 2: Table S2. — OD450 values of ELISA tests for H. pylori-specific IgG and SIgA levels. (DOC 60 kb) [file 12896_2017_345_MOESM2_ESM.doc]

**Table S2.** OD_450_ values of ELISA tests for *H. pylori*-specific IgG and SIgA levels.

| Group | Mouse ID | OD_450_ | |
| --- | --- | --- | --- |
|  |  | Serum IgG | Intestinal SIgA |
| Lpp20 | L-1 | 0.583 | 0.727 |
|  | L-2 | 0.872 | 0.360 |
|  | L-3 | 0.919 | 0.321 |
|  | L-4 | 0.530 | 0.623 |
|  | L-5 | 0.629 | 0.450 |
|  | L-6 | 0.612 | 0.438 |
|  | L-7 | 0.519 | 0.492 |
|  | L-8 | 0.534 | 0.492 |
|  | L-9 | 0.790 | 0.516 |
|  | L-10 | 0.745 | 0.338 |
| Lpp20+LTB | LL-1 | 0.479 | 1.063 |
|  | LL-2 | 0.608 | 0.666 |
|  | LL-3 | 0.627 | 0.715 |
|  | LL-4 | 0.572 | 0.685 |
|  | LL-5 | 0.528 | 0.600 |
|  | LL-6 | 0.443 | 0.755 |
|  | LL-7 | 0.479 | 0.416 |
|  | LL-8 | 0.514 | 0.614 |
|  | LL-9 | 0.482 | 1.005 |
|  | LL-10 | 0.601 | 0.861 |
| PBS | P-1 | 0.318 | 0.400 |
|  | P-2 | 0.412 | 0.648 |
|  | P-3 | 0.517 | 0.559 |
|  | P-4 | 0.455 | 0.373 |
|  | P-5 | 0.340 | 0.467 |
|  | P-6 | 0.331 | 0.370 |
|  | P-7 | 0.300 | 0.296 |
|  | P-8 | 0.339 | 0.066 |
|  | P-9 | 0.492 | 0.428 |
|  | P10 | 0.347 | 0.406 |
